# Peri‐operative surgical care optimisation for patients requiring emergency surgery (PERI‐SCOPES): Protocol for a multicentre mixed‐methods study

**DOI:** 10.1111/codi.70637

**Published:** 2026-11-05

**Authors:** Rachel John‐Charles, Joanne McPeake, Carly Bisset, Ellen Groundwater, Jennifer Cleland, Tara Quasim, Susan Moug

**Affiliations:** ^1^ Department of Surgery Royal Alexandra Hospital Paisley UK; ^2^ University of Glasgow Glasgow UK; ^3^ The Healthcare Improvement Studies Institute University of Cambridge Cambridge UK; ^4^ Department of Surgery Glasgow Royal Infirmary Glasgow UK; ^5^ Lee Kong Chian School of Medicine, Nanyang Technological University Singapore and National Healthcare Group Singapore Singapore; ^6^ Department of Critical Care Glasgow Royal Infirmary Glasgow UK; ^7^ Department of Surgery Golden Jubilee National Hospital Clydebank UK

**Keywords:** decision‐making, emergency general surgery, quality of life, survivorship

## Abstract

**Introduction:**

Emergency General Surgery is a common reason for an emergency admission in the UK with over 25,000 emergency laparotomies (EmLap) performed every year.(1) For frail and co‐morbid patients an alternative decision is often made that not undergoing surgery is in their best interest (NoLap). For both EmLap and NoLap patients, little is known about survivorship. This study aims to quantify quality of life (QoL) for EmLap and NoLap patients and explore the decision‐making process.

**Methods and Analysis:**

PERI‐SCOPES is a mixed‐methods multicentre study exploring QoL and decision‐making in Emergency General Surgery. The study will involve two separate workstreams.

**Workstream 1:**

Up to 30 patients, EmLap or NoLap, will be eligible for inclusion and identified by the local research teams. Patients will be recruited from six sites across Scotland. Patients will be invited to nominate a family member/supporter to participate alongside them. Participants will complete validated QoL questionnaires within 4 weeks of EmLap/NoLap decision then again at 3‐ and 9–12 months post‐decision. Questionnaires will include EuroQol five‐dimension five‐level, Short Form of the Profile of Mood States, International Trauma Questionnaire and Caregiver Strain Index. Patients and their family member/supporter will also take part in three semi‐structured interviews at similar time‐points to explore their experience of decision‐making.

**Workstream 2:**

UK‐based consultant decision‐makers (surgeons, anaesthetists and intensivists) will be invited to complete an online survey exploring personality, risk‐taking and decision‐making in Emergency General Surgery. At least 52 consultants will be recruited to ensure sufficient statistical power. Individual semi‐structured interviews will be conducted with up to 20 consultants to allow a deeper exploration of decision‐making.

**Ethics and Dissemination:**

PERI‐SCOPES is sponsored by The University of Glasgow and has received ethical approval from East of Scotland REC (25/ES/0015). This study has Research and Innovation (R&I) approval from NHS Greater Glasgow and Clyde, Grampian, Tayside and Lothian (6 hospital sites). We aim to disseminate results via peer‐reviewed medical journals and conferences.

**Trials Registration Number:**

NCT06972017.

## BACKGROUND

Emergency General Surgery (EGS) is an umbrella term which describes all patients presenting to hospital with an acute abdominal pathology. This includes a wide range of benign and cancer‐related conditions which can occur at any age. EGS is a common reason for an emergency admission in the UK with over 25,000 emergency laparotomies (EmLap) performed every year [1, 2].

In Scotland, there is an established national EGS database called Emergency Laparoscopic and Laparotomy Scottish Audit (ELLSA) which was inspired by National Emergency Laparotomy Audit (NELA) in England and Wales [1, 2]. Data collection from 16 acute sites in Scotland has consistently shown mortality in EGS to be five times higher than in elective general surgery [2] The 2022 ELLSA report showed a 30‐day mortality rate of 10.8%, making reduction in mortality rates a key priority [2, 3]. Currently, survivorship or long term outcomes after undergoing high‐risk EGS remains unclear with many EmLap patients likely to struggle with frailty, new multi‐morbidity and lower Quality of Life (QoL) [4]. In support of this statement, a single‐centre study in Scotland revealed that nearly half of patients felt that their operation still impacted their daily activities 1 year later [4].

There is another patient population in EGS, one that is consistently overlooked: the NoLap (‘No Laparotomy’) population. NoLap patients are often complex, frail, multi‐morbid, older adults with pathology that needs surgery, but the decision is made that undergoing surgery is not in their best interest and will likely cause more harm. NELA began collecting data for NoLap patients with bowel perforation and ischaemia in April 2024, however other pathologies are not yet recorded. [5, 6] ELLSA began collecting NoLap data in October 2025. In the largest UK study to date, from 750 NoLap patients 21% survived at least 90‐days, but no studies have investigated QoL in this population [7].

The importance of QoL and its potential impact on decision‐making is evolving. A recent patient and public involvement (PPI) study showed that a decline in post‐operative QoL would influence a patient's decision to have emergency surgery more than risk of mortality [8]. This emphasises the importance of accurately understanding post‐operative QoL. A systematic review published in 2024 identified 11 studies comparing QoL post‐EmLap with a comparator group between 2000 and 2023 [9]. Overall, post‐operative QoL was lower than that of the comparison group in eight studies and most patients reported lower QoL for at least 3 months [9]. However, the average age was 61 years; the authors recognised this as a limitation considering older patients are often more co‐morbid and frail impacting recovery and likely QoL. Another systematic review published in 2023 only identified 11 clinical trials investigating QoL post‐EmLap between 2011 and 2020 [10]. Both these reviews highlighted the sparsity of long‐term QoL research and emphasised the need for dedicated EGS Patient‐Reported Outcome and Experience Measures (PROMs/PREMs) [9].

Decision‐making in elective general surgery has been widely explored; however, there is limited research dedicated to EGS [11]. In the emergency setting, decision‐making can be more challenging [8] Patients are often unprepared, distressed, in pain, and on sedating medication, making it difficult to retain information, balance risk and make an informed decision [8] Often, these decisions must be made quickly, and clinicians need to provide enough information to allow patients/families to make informed decisions whilst not overloading them with information [8] The way clinicians convey information is crucial to allow patients to make appropriate decisions which align with their treatment goals [12].

Expanding our knowledge of EmLap and NoLap decision‐making from patients, families/supporters and clinicians' perspectives will highlight areas for possible clinical improvement and the associated development and implementation of tailored training and education.

## OBJECTIVES

The primary objectives of the study are to [1] report on the long‐term QoL of EmLap and NoLap patients and their families/supporters and [2] explore decision‐making in EGS from the perspective of patients, families/supporters and clinicians.

## METHODS AND ANALYSIS

### Study design and setting

PERI‐SCOPES is a multicentre mixed‐methods prospective cohort study which will be conducted and reported in line with the guidance presented in the Strengthening and Reporting of Observational Studies in Epidemiology (STROBE) statement. [13] The consolidated criteria for reporting qualitative research (COREQ) checklist will be used for the qualitative work, whilst the checklist for reporting results of internet e‐surveys (CHERRIES) will be used for online survey. [14, 15].

The study has two workstreams:

### Workstream 1

- This workstream will quantify QoL following EmLap/NoLap decision and explore participant experiences of decision‐making in EGS.
- Participants will include EmLap and NoLap patients and their families/supporters
- Participants will complete validated QoL questionnaires and semi‐structured interviews within 4 weeks of EmLap/NoLap decision then again at 3‐ and 9–12 months post‐decision.
- Where completion of questionnaires and interviews within 4 weeks of the EmLap/NoLap decision is not feasible, the research team will undertake these assessments at the earliest possible opportunity, aiming to complete them as close to the specified timeframe as possible.
- This follow‐up period was designed to capture the immediate impact of the decision, as well as short‐term and long‐term effects
- During workstream 1, the specialty of consultants involved in the decision‐making process will be recorded to inform the inclusion criteria for workstream 2.

### Workstream 2

- This workstream will explore consultant decision‐making in EGS through an online survey and semi‐structured interviews.
- The survey will involve personality testing, risk‐taking scoring and decision‐making for difficult hypothetical EGS scenarios. The interview will allow a deeper exploration of decision‐making.
- The specialities involved in consultant decision‐making will be identified in workstream 1 – likely to be surgeons, anaesthetists and intensivists.

### Participants

This study involves three groups: patients, families/supporters and consultants (surgeons, anaesthetists and intensivists).

Workstream 1 will be conducted at six NHS hospitals in Scotland: Queen Elizabeth University Hospital, Glasgow Royal Infirmary, Royal Alexandra Hospital, Aberdeen Royal Infirmary, Ninewells Hospital and Edinburgh Royal Infirmary. These hospitals perform the highest number of EmLaps per year in Scotland as reported in ELLSA [2].

#### Group 1: Patients (up to 30 participants)

##### Inclusion criteria

- Age ≥ 65 years old (older patients are often more co‐morbid and frail impacting recovery and QoL)
- Able to communicate in English
- Cognitively able to complete the survey/interviews
- Able to provide informed, voluntary consent
- First line treatment is expedited, urgent or emergency abdominal surgery on the gastrointestinal tract
- Surgery can be laparoscopic or open approach.
- Inclusion criteria for EmLap should mirror NELA inclusion criteria. [16] (Data S1)
- Inclusion criteria for NoLap should mirror the inclusion criteria detailed in ELF‐2 (Emergency Laparotomy and Frailty Study 2). ELF‐2 was a UK‐based multi‐centre cohort study which recruited 750 NoLap patients in 2021. [7] This study will replicate their inclusion criteria considering it is the largest NoLap study to date in the UK [7].

##### Exclusion criteria

- Age < 65 years old
- NoLaps should be excluded if management involved interventional radiology or endoscopic procedures
- Patients who are offered a period of conservative treatment are not automatically NoLap if surgery may ultimately be offered
- Diagnosis of dementia or long‐standing cognitive impairment
- Elective laparotomy/laparoscopy
- Diagnostic laparotomy/laparoscopy where no subsequent procedure is performed. However, if no procedure is performed because of inoperable pathology, then the patient can be included
- All surgery involving the appendix or gallbladder, including any surgery relating to complications
- Non‐elective hernia repair without bowel resection or division of adhesions.
- Non‐elective formation of colostomy or ileostomy
- Trauma surgery (blunt or penetrating), vascular surgery, obstetric/gynaecological surgery or transplant surgery
- Surgery for pathology of oesophagus, spleen, renal tract, kidneys, liver, gallbladder and biliary tree, pancreas or urinary tract

#### Group 2: Families/supporters (up to 30 participants)

##### Inclusion criteria

- Identified by the patient participant (only approached if nominated by patient)
- Age ≥ 18 years old
- Able to communicate in English
- Cognitively able to complete the survey/interviews
- Able to provide informed, voluntary consent

##### Exclusion criteria

- Age < 18 years old
- Lack of patient consent to family/supporter involvement

#### Group 3: Consultants (interview: 12–20 participants) (survey: Minimum 52 participants)

##### Inclusion criteria

- The specialities of the key decision‐makers will be identified in workstream 1 (likely: surgeons, intensivists and anaesthetists)
- Post‐CCT (Certificate of Completion of Training)
- Participate in active on‐call for EGS in a UK‐based Hospital where they make decisions in EGS regularly

##### Exclusion criteria

- Consultants with less than 2 years of post‐CCT on‐call experience

### Primary aim

The primary aim of workstream 1 is to report on the long‐term QoL of EmLap and NoLap patients and their families/supporters. For this, participants will complete validated QoL questionnaires within 4 weeks of an EmLap/NoLap decision then again at 3‐ and 9–12‐months post‐decision. These are summarised in Table 1. Using a variety of tools over 1 year will provide a detailed insight into QoL during the recovery journey.

**TABLE 1:** Participant timeline for follow‐up questionnaires.

Questionnaires	Patient's			Families/supporters		
	Within 4 weeks	3 months	9 to 12 months	Within 4 weeks	3 months	9 to 12 months
EuroQol five‐dimension five‐level (EQ‐5D‐ 5 L)	x	x	x	x	x	x
Short form of the Profile of Mood States (SF‐POMS)	x	x	x	x	x	x
International Trauma Questionnaire (ITQ)		x	x		x	x
Caregiver Strain Index (CSI)					x	x

The primary aim of workstream 2 is to describe and explore decision‐making in EGS by the key consultant decision‐makers through an online survey and semi‐structured interviews.

### Secondary aims

Workstream 1 will clarify which specialties were involved in EGS decision‐making to help recruitment for workstream 2. We expect the inclusion of surgeons, anaesthetists and intensivists. The research team behind ELF‐2 are also exploring which clinicians are involved in the EmLap/NoLap decision and their results may influence workstream 2 recruitment [7].

The results from workstream 1 will inform the development of PROMs/PREMs for EGS. There is currently no QoL data collection or PROMs/PREMs incorporated into ELLSA. Current literature demonstrates that the use of PROMs/PREMs in EGS is feasible; however, EGS specific questionnaires are needed. [17].

The online survey in workstream 2 will also explore possible associations between clinician personality/risk‐taking and decision‐making in EGS. Previous research has explored this topic; however, this work was largely focused on elective general surgery [18, 19]. Improving our understanding of the role of personality in clinical practice is fundamental to establishing its possible impact.

### Participant timeline

#### Workstream 1

##### Baseline assessments

Patient baseline characteristics will be collected, including EmLap/NoLap decision date, demographics, co‐morbidities, NELA score, clinical frailty score, clinical/radiological diagnosis and social/functional circumstances. Information will be recorded by the local research team in a patient Case Report Form (CRF) (Data S2). Patients will be given a diary to record any experiences of hospital visits/appointments attended after discharge. Family/supporter demographics will be recorded at the time of recruitment in a family/supporter CRF (Data S3).

##### Follow‐up questionnaires

Questionnaires will be posted to participants within 4 weeks of EmLap/NoLap decision then again at 3‐ and 9‐12‐months post‐decision. These questionnaires are described in Table 1. Participants will be asked to return questionnaires in a prepaid and addressed envelope to Royal Alexandra Hospital (principal research site).

There is currently no validated PROM to measure QoL following EGS admission. Therefore, following literature review and expert group discussion, the following validated questionnaires were selected by an experienced team of EGS researchers. EuroQol five‐dimension five‐level (EQ‐5D‐5L) is a well‐regarded, simple and widely utilised QoL tool in healthcare research [10]. Short form of the Profile of Mood States (SF‐POMS) is a commonly used psychometric tool which measures the mood states of tension, depression, anger, vigour, fatigue and confusion [20]. International Trauma Questionnaire (ITQ) assesses the affect a specific ‘trauma’ (i.e., EGS admission) has on a individuals physical and mental state [21]. Finally, Caregiver Strain Index (CSI) is a well‐designed questionnaire which measures the psychological, social and physical effects of caring on the caregiver. [22] The authors judged these questionnaires appropriately measure aspects of physical, mental and social well‐being commonly impacted following EGS. All questionnaires are already widely applied in clinical research and simple to complete. All relevant permissions have been granted to allow use of these questionnaires.

##### Semi‐structured interviews

Interviews will be conducted by study co‐ordinator (RJC) and will last between 30 and 60 min. Patients will be interviewed with their family member/supporter unless participants request the interviews to be carried out separately. Interviews will either be face‐to‐face or virtual via secure NHS videoconferencing, depending on the participants' preference. All interviews will be recorded and transcribed. Recordings will be kept for future presentations at conferences if explicit consent has been given by participants. A semi‐structured interview approach will allow exploration of specific themes such as decisional regret/conflict and the decision‐making experience (Data S4) [23, 24]. However, we hope this interview technique will also allow some flexibility for participants to explore other areas of interest. Repeated interviews over 1 year (longitudinal qualitative research) will allow a deeper exploration of participants' experiences and how these may change as they progress through their recovery.

#### Workstream 2

##### Baseline assessments

Consultant baseline characteristics will include gender, sub‐speciality, deanery of current practice and years of consultant experience. This information will be documented in a consultant CRF (Data S5).

##### Online survey

The online survey will include personality testing (Big 5 Inventory) and risk‐taking scoring (30‐item Domain Specific Risk‐taking scale) [25, 26]. Followed by 20 hypothetical EGS scenarios developed from Workstream 1 which will explore difficult EmLap/NoLap decisions where there is currently no evidence to determine the ‘best’ course of action. This survey will be created using SNAP 11.

##### Semi‐structured interviews

Interviews will be conducted by trained clinical researchers within the PERI‐SCOPES study team and will last between 30 and 60 min. Discussions will explore experiences of EGS decision‐making, difficult decisions in the palliative setting, decisional regret and perceptions of the impact of personality. Workstream 1 and Workstream 2 online surveys will inform the development of this interview schedule.

### Sample size

#### Workstream 1

We aim to recruit up to 30 patients (group 1) and 30 family members/supporters (group 2). This equates to up to five patients and five family members/supporters from each hospital. Previous literature shows that this number should generate sufficient thematic data for meaningful qualitative analysis (i.e., information power). [27] Families/supporters play an integral role in a patient's hospital experience and recovery, therefore exploring their perspectives is important. Furthermore, due to the high‐risk nature of EGS, families/supporters will be offered the opportunity to remain in the study if the patient withdraws.

#### Workstream 2

We aim to have a minimum of 52 consultants (group 3) complete the online survey. Recent work found that the average association between personality and decision making is 0.37 [18]. Using this effect size estimate to achieve a power of 0.80, with α (2‐tailed) of 0.05 requires 52 participants.

For consultant interviews, we aim to recruit between 12 and 20 participants. This sample size is judged appropriate given these are targeted participants with expertise discussing a specific research question. [28].

We will stop interviewing when data saturation is reached, that is, no new themes emerge from ongoing interviews [28]. Normally, when no new themes emerge after three consecutive interviews. This decision will be made by the primary analysis team (RJC, JM and SM).

### Recruitment

#### Workstream 1

Patients can be recruited within 4 weeks of EmLap/NoLap decision. Purposive sampling will be used to encourage relatively equal numbers of EmLap and NoLap patients, as well as recruitment from all six hospitals to ensure geographical diversity. Sociodemographic diversity will be achieved by seeking participants with various educational backgrounds and different employment statuses.

Appropriate patients will be approached by the local clinical team to discuss the study and provide the participant information sheet (PIS; Data S6). Patients can nominate family members/supporters to be approached if they wish. The family member/supporter will also be given a PIS (Data S7). A patient can still be recruited even if they do not wish or cannot identify a family member/supporter. Participants will be given a minimum of 24 h to consider. If interested, the study co‐ordinator (RJC) will contact them to discuss further, complete consent (Data S8 and S9) and finalise enrolment.

#### Workstream 2

Recruitment will involve purposive sampling with advertisement through established professional networks and in relevant conferences. Interested consultants will receive PIS via email (Data S10 and S11). The start of the online survey will incorporate an electronic consent form (Data S12). Electronic consent forms will be emailed to interview participants to complete (Data S13).

Purposive sampling will be used to ensure consultants from each specialty are recruited (surgeons, intensivists and anaesthetists). Recruitment of new participants will continue until data saturation is reached in the interviews (i.e., when no new themes emerge after three consecutive interviews). This may mean different numbers of participants from each speciality.

### Data collection

Participants will be assigned a participant identification number (PID). Their corresponding research data will be coded with the same PID to ensure anonymity. All data will be collected, handled and stored in accordance with the Data Protection Act (2018).

Local research teams will collect baseline characteristics for Group 1 by reviewing electronic healthcare records. Participants will complete their own questionnaires/surveys to minimise bias. If there is a delayed response to follow‐up requests, the research team will contact participants a maximum of twice before deeming ‘lost to follow‐up’.

### Data management

Data management, processing and handling will be conducted in line with General Data Protection regulation principles (EU 2016/679).

PID and corresponding personal identifiable information will be stored on password‐protected encrypted NHS computer with restricted access. Personal identifiable information will be securely deleted 1 year after the study ends.

The research team will transcribe data from paper‐based CRFs and questionnaires onto electronic datasheets on a password‐protected encrypted NHS computer with restricted access. Interview transcripts will be stored similarly. We aim to carry out an immediate transcription and anonymisation process with pseudonyms for participants. At the end of the study, anonymous research data will be stored for 10 years, in line with The University of Glasgow's policies and procedures.

### Data analysis

Validated questionnaires will be scored in accordance with their scoring manuals. Missing data will be handled using mean substitution.

Baseline demographics will be reported using descriptive statistics. Parametric data will be described using mean and SE of mean; non‐parametric data will be described using median and IQR; categorical data will be described in percentages. Statistical significance will be assessed using CHI‐squared testing for categorial variables; one‐way ANOVA for parametric continuous variables; Mann–Whitney U for non‐parametric continuous data with two variables and Kruskal–Wallis if more than two variables. Multivariate analysis will investigate associations between the various outcomes. *p* values <0.05 will be considered statistically significant.

Participant interviews will be recorded and transcribed using secure NHS videoconferencing. Qualitative analysis will follow the Braun and Clarke six‐step framework using reflexive thematic analysis with an inductive approach [29]. These steps include familiarisation of the data; generating initial codes, combining codes into themes, reviewing themes, defining and naming themes, and finally, reporting the findings [29]. There will be iterative discussion across the six steps of the thematic analysis between two researchers. Any disagreements will be discussed and resolved with the wider research team. The primary analysis team will then extract relevant quotes to support the thematic analysis and the primary findings of the study. An audit trail will be maintained to demonstrate and delineate decision making. Interview data will be managed in NVivo qualitative data analysis software.

### Harm

We anticipate minimal risk to participants as a result of taking part in this study. However, we understand that exploring their experiences during the interview may be upsetting. Interviews will be conducted by an experienced surgical trainee (study co‐ordinator; RJC) who has first‐hand experience dealing with difficult scenarios in EGS. Participants will be encouraged to postpone or withdraw if participation becomes distressing. Referral to suitable support services will be organised if felt appropriate.

### Patient and public involvement

Previous PPI research by this study's chief investigator (SM) and co‐investigator (CB) has highlighted the importance of QoL to patients when making decisions about EmLap. [8] This work led to the development of PERI‐SCOPES.

A representative from Bowel Research UK (BRUK) People and Research Together (PaRT) network was involved during PERI‐SCOPES inception, design and grant application process. More BRUK PaRT representatives will be invited to join the study steering group.

### Ethics and dissemination

PERI‐SCOPES is sponsored by The University of Glasgow and has received a favourable opinion by the East of Scotland Research and Ethics Committee (25/ES/0015). This study has Research and Innovation (R&I) approval from NHS Greater Glasgow and Clyde, Grampian, Tayside and Lothian.

Any amendments to the protocol will be submitted to the appropriate ethics committee via the Integrated Research Application System (IRAS) Amendment Portal and NHS R&I for approval prior to implementation. All protocol amendments will be cited and explained in future work.

All participants will be required to complete written consent prior to enrolment in the study. During data collection, personal identifiable information will be pseudonymised to maintain confidentiality. Paper‐based consent forms and questionnaire responses will be stored separately in a locked NHS office with restricted access. Personal information and electronic research data will be stored on separate databases on encrypted and password‐protected NHS computers with restricted access. Only essential members of the PERI‐SCOPES research team will have access to the final dataset.

Study results will be disseminated through publication in peer‐reviewed medical journals and presentation at medical conferences. Participants can opt to receive results in a study newsletter if they wish.

## CONCLUSION

PERI‐SCOPES is a mixed‐methods multicentre study which will explore QoL and decision‐making in EGS through two workstreams involving patients, family members/supporters and clinicians.

Despite being a significant issue worldwide, EGS remains under‐researched. [30] National initiatives, such as NELA and ELLSA, have demonstrated the benefit of EGS research, which is reflected in improving patient outcomes [30]. However, despite these improvements, there is still scope for further research.

## AUTHOR CONTRIBUTIONS

**Rachel John‐Charles:** Writing – original draft; methodology; writing – review and editing; project administration; conceptualization. **Joanne McPeake:** Writing – review and editing; methodology; supervision; conceptualization. **Carly Bisset:** Conceptualization; writing – review and editing; methodology; supervision. **Ellen Groundwater:** Methodology; project administration; conceptualization. **Jennifer Cleland:** Writing – review and editing; methodology; supervision; conceptualization. **Tara Quasim:** Methodology; writing – review and editing; supervision; conceptualization. **Susan Moug:** Conceptualization; funding acquisition; writing – review and editing; methodology; project administration; supervision.

## FUNDING INFORMATION

PERI‐SCOPES has received funding from Bowel Research UK (BRUK). Grant number PhD_23017. BRUK will not have responsibility or final decision for study design, conduct, data analysis and interpretation, manuscript writing and dissemination of results.

## CONFLICT OF INTEREST STATEMENT

The authors declare no conflicts of interest.

## ETHICS STATEMENT

PERI‐SCOPES is sponsored by The University of Glasgow and has received ethical approval from East of Scotland REC (25/ES/0015).

## Supporting information


Data S1:

Data S2:

Data S3:

Data S4:

Data S5:

Data S6:

Data S7:

Data S8:

Data S9:

Data S10:

Data S11:

Data S12:

Data S13:

## Data Availability

The data that support the findings of this study are available from the corresponding author upon reasonable request.
