# Supplementary material for: Peri‐operative surgical care optimisation for patients requiring emergency surgery (PERI‐SCOPES): Protocol for a multicentre mixed‐methods study
Source: Colorectal Dis. 2026 Nov 5;28(11):e70637. doi: 10.1111/codi.70637 (PMC13740466; doi:10.1111/codi.70637)
Supplement: Supplementary file 1 [file CODI-28-0-s001.docx]

**Supplementary Materials**

National Emergency Laparotomy Audit (NELA) Inclusion and Exclusion Criteria.

| **NELA** | Aged 18 y and over |
| --- | --- |
| **Inclusion** | Open, laparoscopic, or laparoscopically assisted procedures  Procedures involving the stomach, small or large bowel, or rectum  For conditions such as perforation, ischemia, abdominal abscess, bleeding, or obstruction  Washout/evacuation of intraperitoneal abscess (unless due to appendicitis or cholecystitis—excluded, see below)  Washout/evacuation of intraperitoneal hematoma  Bowel resection/repair due to incarcerated incisional, umbilical, inguinal, and femoral hernias (but not hernia repair without bowel resection/repair). Bowel resection/repair due to obstructing/incarcerated incisional hernias provided the presentation and findings were acute. This will include large incisional hernia repair with division of adhesions.  Laparotomy/laparoscopy with inoperable pathology (eg, peritoneal/hepatic metastases) where the intention was to perform a definitive procedure. This does not include purely diagnostic procedures  Laparoscopic/Open adhesiolysis  Return to theatre for repair of substantial dehiscence of major abdominal wound (ie, ‘‘burst abdomen’’)  Any reoperation/return to theatre for complications of elective general/upper GI surgery meeting the criteria above is included. Returns to theatre for complications following non-GI surgery are now excluded (see exclusion criteria below). If multiple procedures are performed on different anatomical sites within the abdominal/pelvic cavity, the patient would be included if the major procedure is general surgical. For example, nonelective colonic resection with hysterectomy for a fistulating colonic cancer would be included as the bowel resection is the major procedure  However, bowel resection at the same time as emergency abdominal aortic aneurysm repair would not be included as the aneurysm repair is the major procedure |
| **NELA** | Patients under 18 |
| **Exclusion** | Elective laparotomy/laparoscopy  Diagnostic laparotomy/laparoscopy where no subsequent procedure is performed (NB, if no procedure is performed because of inoperable pathology, then include) |

Appendicectomydrainage of localized collection unless the procedure is incidental to a nonelective procedure on the GI tract

Cholecystectomydrainage of localized collection unless the procedure is incidental to a nonelective procedure on the GI tract (All surgery involving the appendix or gallbladder, including any surgery relating to complications such as abscess or bile leak is excluded. The only exception to this is if carried out as an incidental procedure to a more major procedure. We acknowledge that there might be extreme cases of peritoneal contamination, but total exclusion avoids subjective judgementjudgment calls about severity of contamination.)

Minor abdominal wound dehiscence unless this causes bowel complications requiring resection

Nonelective formation of a colostomy or ileostomy as either a trephine or a laparoscopic procedure (NB: if a midline laparotomy is performed, with the primary procedure being formation of a stoma, then this should be included) Vascular surgery, including abdominal aortic aneurysm repair

Caesarean section or obstetric laparotomies; gynaecological laparotomy; ruptured ectopic pregnancy, or pelvic abscesses due to pelvic inflammatory disease

Laparotomy/laparoscopy for pathology caused by blunt or penetrating trauma

All surgery relating to organ transplantation (including returns to theatre for any reason following transplant surgery)

Surgery relating to sclerosing peritonitis

Surgery for removal of dialysis catheters

Laparotomy/laparoscopy for oesophageal pathology

Laparotomy/laparoscopy for pathology of the spleen, renal tract, kidneys, liver, gall bladder and biliary tree, pancreas, or urinary tract

Returns to theatre for complications (eg, bowel injury, hematoma, collection) following non-GI surgery are now excluded, that is, returns to theatre following renal, urological, gynaecological, vascular, hepatic, pancreatic, splenic surgery are excluded.
