# Supplementary material for: Peri‐operative surgical care optimisation for patients requiring emergency surgery (PERI‐SCOPES): Protocol for a multicentre mixed‐methods study
Source: Colorectal Dis. 2026 Nov 5;28(11):e70637. doi: 10.1111/codi.70637 (PMC13740466; doi:10.1111/codi.70637)
Supplement: Supplementary file 2 [file CODI-28-0-s002.docx]

# **PERI-SCOPES**

**Peri-Operative Surgical Care Optimisation for Patients requiring Emergency Surgery**

**FAMILY/SUPPORTER CRF**

**PERI-SCOPES PARTICIPANT ID:**

- Family/supporter CRF to be completed by member of research team (research nurse/RJC)
- Baseline measurements: at time of recruitment.
- Assessment 1: within 4 weeks of decision date
- Assessment 2: 3 months after decision date
- Assessment 3: between 9-12 months after decision date
- Questionnaires: within 4 weeks, 3 months, between 9-12 months

**Participant Eligibility**

**Inclusion**

- Identified by the patient participant
- Age ≥18 years old
- Able to communicate in English
- Cognitively able to complete the survey/interviews
- Able to consent

**Consent Details**

Date of consent:

Consent version:

Consented by:

| **Baseline Measurements** *(time of recruitment)*  Date of Assessment:  Completed by: (Researcher name) | | |  | |
| --- | --- | --- | --- | --- |
| **Section One: Demographics & Basic information** | | | | |
| 1.1 | Sex | 🞏 Male | | 🞏 Female |
| 1.2 | Age at time of recruitment |  | | |
| 1.3 | Scottish Deprivation Score | Overall rank/Decile | | |
| 1.4 | Education level |  | | |
| 1.5 | Ethnicity |  | | |
| 1.6 | Work status | 🞏 Employed  🞏 Unemployed | | 🞏 Retired |
| **Section Two: Medical information** | | | | |
|  |  |  | | |
| 2.1 | Charlson co-morbidity index | Score: | | |
|  |  | Co-morbidities: | | |

| **Questionnaires** | | |  | |
| --- | --- | --- | --- | --- |
| 1.1 | Within 4 weeks of decision date | Date Sent:  Date Returned: | |  |
| 1.2 | 3 months after decision date | Date Sent:  Date Returned: | |  |
| 1.3 | Between 9-12 months after decision date | Date Sent:  Date Returned: | |  |
