# Supplementary material for: Peri‐operative surgical care optimisation for patients requiring emergency surgery (PERI‐SCOPES): Protocol for a multicentre mixed‐methods study
Source: Colorectal Dis. 2026 Nov 5;28(11):e70637. doi: 10.1111/codi.70637 (PMC13740466; doi:10.1111/codi.70637)
Supplement: Supplementary file 4 [file CODI-28-0-s004.docx]

# **PERI-SCOPES**

**Peri-Operative Surgical Care Optimisation for Patients requiring Emergency Surgery**

**Workstream 1 Interview Questions**

- What was your experience of the EmLap or NoLap decision-making process?
- Are you happy with how the EmLap/NoLap decision was made?
- Do you feel like the correct decision was made?
- How do you feel since leaving hospital?
- Any problems since discharge?
- Are you able to carry out your usual activities again?
- Able to carry out personal care?
- Has your mood been affected?
- Has your experience affected your relationships with family or friends?
- How much support is the family member/friend needing to provide?
- What would help the recovery at home?
- Is this recovery what you expected?
- Do you feel like your getting enough support?
