# Supplementary material for: Peri‐operative surgical care optimisation for patients requiring emergency surgery (PERI‐SCOPES): Protocol for a multicentre mixed‐methods study
Source: Colorectal Dis. 2026 Nov 5;28(11):e70637. doi: 10.1111/codi.70637 (PMC13740466; doi:10.1111/codi.70637)
Supplement: Supplementary file 6 [file CODI-28-0-s006.docx]

# **PERI-SCOPES**

**Peri-Operative Surgical Care Optimisation for Patients requiring Emergency Surgery**

**PARTICIPANT INFORMATION SHEET**

**A study exploring consultant decision-making in emergency general surgery**

**Invitation**

Before you decide whether to take part, it is important for you to understand why the research is being done, and what it would involve. Please take time to read the following information carefully. Please ask the research team if there is anything that is not clear, or if you would like more information. If you decide to take part in this study, you will be given a copy of this Participant Information Sheet and electronic consent form. Thank you for taking the time to read this.

**Why are we doing this study?**

Emergency General Surgery (EGS) is an umbrella term which describes all patients presenting to hospital with an acute abdominal pathology. EGS is one of the most common reasons for an emergency admission in the UK with over 25,000 emergency laparotomies performed every year. Morbidity and mortality in EGS is significantly higher than in elective general surgery. As a result, EGS is often referred to as ‘high-risk surgery’ and for those patients that have significant co-morbidity, decisions can be made that undergoing surgery is not in their best interests. This group is referred to as NoLaps.

This research will explore how consultants make this important decision about whether to ‘operate’ or ‘not operate’. Your involvement in this study would involve completing an online survey. We are asking consultants from various specialities to be involved – general surgeons, anaesthetists and intensivists.

Expanding our knowledge of EmLap and NoLap decision-making from consultants’ perspectives will highlight areas for possible improvement allowing development of new training and education tools for the future.

**Why am I being asking to take part?**

We are asking consultants who participate in an active on-call for EGS in a UK-based Hospital where they make decisions in EGS regularly. We are hoping to recruit consultants from general surgery, anaesthetics and critical care.

**Do I have to take part?**

No, it is up to you to decide whether or not to take part. If you do decide to take part, you will be given this information sheet to keep and be asked to complete electronic consent. If you decide to take part, you are still free to withdraw at any time and without giving a reason.

**What would taking part involve?**

If you agree to take part you will be asked to complete an online survey. The survey will include:

- Electronic consent (completed version will be emailed to you for your records).
- Demographics – age, gender, deanery of current practice, sub-speciality, years of experience
- Big Five Inventory (44-item validated personality test)
- 30-item DOSPERT (Domain Specific Risk-taking scale)
- Responses to 20 hypothetical emergency general surgery scenarios

During the consent form you will be asked whether you are willing to be contacted in the future to consider participation in an interview exploring similar topic.

**Are there any benefits or risks to taking part?**

There are no anticipated risks from taking part in the study. The main burden of the study is the time taken to complete the online survey. The survey will take approximately 45-60 minutes to complete. You will receive no direct benefit from taking part in this study. However, involvement in the study will contribute to EGS research and expand our knowledge of the complex decision-making process in EGS.

**Who is organising and funding the study?**

The University of Glasgow is the sponsor of this study. They are responsible for the design and conduct of the study. The lead of the study is Professor Susan Moug, Consultant Colorectal Surgeon at Royal Alexandra Hospital in Paisley, Glasgow. The study has received funding from Bowel Research UK. The study has been reviewed and given a favourable ethical opinion by a NHS Research Ethics Committee. (Insert REC details) The study has been given management approval by the Health Boards Research and Innovation department.

**Will my information be confidential?**

All information is confidential and is kept in accordance with the General Data Protection Regulation (GDPR) and the Data Protection Act 2018. All information which is collected about you and responses that you provide during the survey will be kept strictly confidential. You will be identified by a participant ID number, and any information about you will have your name and email address removed so that you cannot be recognised from it. All data will be stored on secure password–protected NHS computer. We will use secure NHS systems to transfer data when needed. Your confidential information (name/email address) will be retained only for as long as is necessary for processing. After this time, personal data will be securely deleted. At the end of the study anonymous data will be stored for 10 years, in line with the University of Glasgow’s policies and procedures.

Please also refer to the Privacy notice on how we process your personal data.

**How will my information be used?**

The University of Glasgow is the sponsor of this research and is responsible for looking after your information. Everyone involved in the study will keep all information about you safe and secure. Only people who need to know your confidential information will be able to access it. Your research data will have a study number instead. People will use this information to do the research or to check your records to make sure that the research is being done properly.

Your research data (survey responses) will be combined with other participants’ data and analysed. We will make sure no-one can work out who you are from the reports we write. We will also keep the anonymous research data for use in future projects. This might involve sharing your data with other researchers – this will not include any of your personal information, so they won’t be able to identify you, contact you or trace the data back to you.

**What are my choices about how my information is used?**

You can stop being part of the study at any time, without giving a reason, but we will keep the information that we already have. For the study to be reliable we need to manage your data in a specific way. This means we won’t be able to let you see or change the data we hold about you.

**What will happen when the study ends?**

The results of this research study are likely to result in publication in a scientific journal or presentation at conferences over the next 3-4 years. Some data will be used as part of a Doctor of Philosophy (PhD) educational qualification. Your personal information will not be disclosed in any results. Circulation of these research outputs can be provided to the participants on request.

**Where can I find out more about how my data is used?**

- By sending an email to study co-ordinator: [rachel.john-charles@nhs.scot](mailto:rachel.john-charles@nhs.scot)
- By sending an email to the sponsor’s (University of Glasgow) data protection officer: [dataprotection@glasgow.ac.uk](mailto:dataprotection@glasgow.ac.uk)

**What if I want to make a complaint?**

- If you would like to make a complaint or would like more information on the complaints process, please contact the Research Regulation and Compliance team at the University of Glasgow: [RRC@glasgow.ac.uk](mailto:RRC@glasgow.ac.uk)

**Contact for further information**

Rachel John-Charles, General Surgery Registrar (West of Scotland) and PhD Student (University of Glasgow) : [rachel.john-charles@nhs.scot](mailto:rachel.john-charles@nhs.scot)

Professor Susan Moug, Consultant Colorectal Surgeon and Study Lead: [susan.moug2@nhs.scot](mailto:susan.moug2@nhs.scot)

**Thank you for taking the time to read this information sheet. If you are happy to complete the online survey – please click on the link below.**

***(Insert link for survey)***
