# Supplementary material for: Peri‐operative surgical care optimisation for patients requiring emergency surgery (PERI‐SCOPES): Protocol for a multicentre mixed‐methods study
Source: Colorectal Dis. 2026 Nov 5;28(11):e70637. doi: 10.1111/codi.70637 (PMC13740466; doi:10.1111/codi.70637)
Supplement: Supplementary file 8 [file CODI-28-0-s008.docx]

# **PERI-SCOPES**

**Peri-Operative Surgical Care Optimisation for Patients requiring Emergency Surgery**

**CONSULTANT CONSENT FORM – ONLINE SURVEY**

Please tick box

1. I confirm that I have read and understood the information sheet (version.......date……..) and privacy notice (version…….date..……) for the above study. I have had the opportunity to consider the information, ask questions and have had these answered satisfactorily.
2. I understand that my participation is voluntary and that I am free to withdraw at any time
   without giving any reason.
3. I understand that data collected during the study, may be looked at by individuals from University of Glasgow, from regulatory authorities or from the NHS Trust, where it is relevant to my taking part in this research.
4. I understand that the information collected about me will be used to support
   other research in the future and may be shared anonymously with other researchers.
5. I understand and agree to the way my data will be collected and processed and that data will be stored for up to 10 years in University archiving facilities in accordance with Data Protection policies and regulations.
6. If I decide to withdraw, or am withdrawn from the study, I agree that the information collected about me up to the point of my withdrawal will be used in the data analysis for this study.
7. I consent to personality testing in this survey.
8. I agree that my name, contact details and data described in the information sheet will be kept for the purposes of this research project.
9. I agree to take part in the above study.

NO

YES

10. (Optional) I would like to receive a newsletter with the results of the study.
11. I agree to being contacted in the future to consider participation in an

NO

YES

interview exploring similar topic
