# Supplementary material for: Peri‐operative surgical care optimisation for patients requiring emergency surgery (PERI‐SCOPES): Protocol for a multicentre mixed‐methods study
Source: Colorectal Dis. 2026 Nov 5;28(11):e70637. doi: 10.1111/codi.70637 (PMC13740466; doi:10.1111/codi.70637)
Supplement: Supplementary file 10 [file CODI-28-0-s010.docx]

# **PERI-SCOPES**

**Peri-Operative Surgical Care Optimisation for Patients requiring Emergency Surgery**

**FAMILY/SUPPORTER INFORMATION SHEET**

**A study exploring your experience during this emergency admission and how it effects your future well-being**

**Invitation**

- Before you decide whether to take part, it is important for you to understand why the research is being done, and what it would involve.
- Please take time to read the following information carefully.
- Discuss with family and friends if you wish.
- It is your choice whether to take part in this study – if you choose not to take part it will not affect the care of your family member/friend.
- Please ask us if there is anything that is not clear, or if you would like more information.

**Summary**

- Patients can be admitted under general surgery for various reasons.
- Some conditions, such as bowel obstruction, require an emergency operation. For some patients, the decision is made to treat the same condition without an operation.
- This study will recruit patients who had an emergency operation and those who did not.
- We are asking patients to nominate a family member or friend to be involved in the study alongside them. This will only be done if the patient wishes.
- The study will involve asking supporters, like yourself, for your opinion and experiences of your family member/friends’ admission and the 1 year after.
- We hope the information learnt in this study will help develop better services and improve the care for future patients and their family/supporters.
- Taking part in this study will not affect the treatment your family member/friend receives.
- You can stop taking part at any time.
- If you express an interest in taking part the study co-ordinator will make contact shortly to discuss further and help you complete the attached consent form.

**Why are we doing this study?**

- Emergency general surgery is one of the most common reasons for an emergency admission in the UK.
- Patients are admitted with various conditions requiring emergency operations.
- The decision “operation” or “no operation” was made during this admission.
- We want to explore your feelings about this “decision-making” experience
- Improving our understanding of patients and their supporters’ experiences of decision-making will help us look for ways to improve the process for others in the future.
- We want to learn more about how patients recover after discharge and how this might affect their family/supporters quality of life
  - Has providing care affected your quality of life?
  - Are you struggling with anxiety or depression?
  - Do you worry about events which happened during their admission?
- Learning more about this will allow us to develop ways to help supporters, like yourself, whilst they care for family members or friends after an emergency admission.

**Why am I being asking to take part?**

- We are asking all willing family members/supporters to take part, if:
  - They are over 18 years old.
  - They can communicate in English
  - Your family member or friend has agreed for us to ask you to take part

**What would taking part involve?**

- A member of the medical team will have already approached you to discuss this study.
- They will have given you this information sheet plus consent form and privacy notice.
- If you express an interest in being involved, the study co-ordinator (Rachel John-Charles) will make contact after 24 hours to discuss further.
- If you agree to take part, the study co-ordinator will help you complete the consent form.
- The study will involve you completing questionnaires and taking part in interviews
- Within 4 weeks of your family member/friends discharge from hospital:
  - Questionnaires will be posted to your home for you to fill in.
  - You will take part in an interview.
- 3 months after your family member/friends hospital stay:
  - Questionnaires will be posted to your home for you to fill in.
  - You will take part in an interview.
- 9 to 12 months after your family member/friends hospital stay:
  - Questionnaires will be posted to your home for you to fill in.
  - You will take part in an interview.
- The questionnaires will ask you questions about your quality of life.
- A member of the research team will call you to organise a time for the interview which is suitable for you.
- The study co-ordinator (Rachel John-Charles) will carry out the interviews.
- During the interview you will discuss the decision-making experience and your general well-being.
- The interview will be carried out in-person or online via Microsoft TEAMS or NHS NearME (this is your choice)
- You and your family member/friend can decide whether to take part in the interviews separately or together. If you feel discussing these topics in front of your family member or friend may be distressing, you may choose to be interviewed separately.
- If you wish, you can continue to take part in the study if your family member/friend drops out (we will check your family member/friend agrees to this)
- Questionnaire will be posted to your home to allow you to complete them in private.
- Questionnaire may take 20 to 30 minutes to complete.
- Interviews will last 30 – 60 minutes.
- Both online and in-person interviews will be recorded via digital audio-recorder or Microsoft TEAMS.

Within 4 weeks of admission

Complete questionnaire

Take part in interview

3 months after admission

Complete questionnaire

Take part in interview

Between 9-12 months after admission

Complete questionnaire

Take part in interview

**Do I have to take part?**

No, it is up to you to decide whether to take part. If you do decide to take part, you will be given this information sheet to keep and be asked to sign a consent form.

The study co-ordinator will discuss the consent process with you and ensure you receive a fully signed consent form. This will be given to you in person or posted to your home.

**Are there any benefits or risks to taking part?**

- There are no anticipated risks from the study. The study does not involve any additional treatment or tests.
- The main burden of the study is the time taken to complete the questionnaires and participate in the interviews.
- Questionnaires will be posted to your home. Pre-paid and addressed envelopes to return questionnaires will be included.
- You can decide if you wish the interviews to be carried out face-to-face or virtual. We are happy to travel to your home to conduct interviews face-to-face if this is your preference. If you opt for face-to-face interviews at NHS site we will reimburse you for any travel.
- The research team will not change anything about your routine medical care or your family member/friends. However, if you raise an issue during the interview, the study co-ordinator may be able to recommend services you can approach about this.

**Can I stop taking part?**

- Yes, you can agree now and then change your mind later.
- You can stop taking part at any point and do not need to give a reason.
- This will not affect the care your family member or friend receives.
- If you stop taking part – the information you have provided up to this point will be kept and analysed (no one will be able to identify you from this information).

**Who is organising and funding the study?**

- The University of Glasgow is the sponsor of this study. They are responsible for the design and conduct of the study.
- The lead of the study is Professor Susan Moug, Consultant Colorectal Surgeon at Royal Alexandra Hospital in Paisley, Glasgow.
- The study has received funding from Bowel Research UK.
- The study has been reviewed and given a favourable ethical opinion by a NHS Research Ethics Committee. (Insert REC details)
- The study has been given management approval by the Health Boards Research and Innovation department.

**Will my information be confidential?**

- All information is confidential and is kept in accordance with the General Data Protection Regulation (GDPR) and the Data Protection Act 2018.
- All participants will be given a unique study number rather than using names or CHI numbers.
- Any information that can identify you will be stored on a secure NHS computer which is password protected. We will use secure NHS systems to transfer data when needed.
- Any audio recordings will be typed up and then deleted when the study ends.
- We would like to use original audio or video recordings for future presentations at research conferences – you will be asked if you are willing for us to do this on the consent form (this is entirely voluntary).
- At the end of the study anonymous data (no one will be able to identify you) will be stored for 10 years, in line with the University of Glasgow’s policies and procedures.
- If you have consented to your audio or video recordings being used for presentations at research conferences then this data will not be anonymous.

**How will my information be used?**

- The University of Glasgow is the sponsor of this research and is responsible for looking after your information.
- Everyone involved in the study will keep all information about you safe and secure.
- We will only use information we need for the research study.
- This information will include:
  - Name
  - Date of Birth
  - Contact details
- Only people who really need to know your confidential information will be able to access it. Your research data will have a study number instead.
- People will use this information to do the research or to check your records to make sure that the research is being done properly.
- Your research data (including interview and questionnaire responses) will be combined with other participants’ data and analysed.
- This will allow us to understand more about supporters’ experiences of a general surgery admission and how it affects them long-term.
- Some data will be used as part of a Doctor of Philosophy (PhD) educational qualification.
- We will make sure no-one can work out who you are from the reports we write.
- We will also keep the anonymous research data for use in future projects. This might involve sharing your data with other researchers – this will not include any of your personal information, so they won’t be able to identify you, contact you or trace the data back to you.
- At the end of the study, we will save questionnaire responses and interview transcripts in case we need to check the results.
- Please also refer to the Privacy Notice on how we process your personal data.

**What are my choices about how my information is used?**

- You can stop being part of the study at any time, without giving a reason, but we will keep the information that we already have.
- For the study to be reliable we need to manage your data in a specific way. This means we won’t be able to let you see or change the data we hold about you.

**What will happens when the study ends?**

- We plan to share the results of the study to help patients, family members/supporters, clinical teams and researchers in the future.
- This will be done through presentations at medical conferences and publications in medical journals.
- Your personal information will not be disclosed in any results.
- We would like to send you a summary of the results by post. Let us know on the consent form if you would like to receive this.

**Where can I find out more about how my data is used?**

- By asking one of the research team
- By sending an email to study co-ordinator: [rachel.john-charles@nhs.scot](mailto:rachel.john-charles@nhs.scot)
- By sending an email to the sponsor’s (University of Glasgow) data protection officer: [dataprotection@glasgow.ac.uk](mailto:dataprotection@glasgow.ac.uk)
- <https://www.hra.nhs.uk/planning-and-improving-research/policies-standards-legislation/data-protection-and-information-governance/gdpr-guidance/templates/template-wording-for-generic-information-document/>

**What if there is a problem?**

- If you have any concerns about the study, you should speak to the research team who will do their best to answer your questions.
- You can contact local research team:
  - [rachel.john-charles@nhs.scot](mailto:rachel.john-charles@nhs.scot)
- You can contact the study co-ordinator: [rachel.john-charles@nhs.scot](mailto:rachel.john-charles@nhs.scot)
- You can contact the study lead: [susan.moug2@nhs.scot](mailto:susan.moug2@nhs.scot)
- You can contact the University of Glasgow (Study Sponsor): [RRC@glasgow.ac.uk](mailto:RRC@glasgow.ac.uk)
- You can contact Patient Advice and Support Service on:
  - 0800 917 2127
  - <https://pass-scotland.org.uk/contact/>
- We have no reason to believe that you will be placed at any greater risk by taking part in this research study. If anything goes wrong because of taking part in the study, there are no special compensation arrangements. If you are harmed and this is due to someone’s negligence then you may have grounds for legal action, but you may have to pay your legal costs. The normal NHS complaints mechanisms will still be available to you.

**Thank you for taking the time to read this information sheet**
