# Supplementary material for: Peri‐operative surgical care optimisation for patients requiring emergency surgery (PERI‐SCOPES): Protocol for a multicentre mixed‐methods study
Source: Colorectal Dis. 2026 Nov 5;28(11):e70637. doi: 10.1111/codi.70637 (PMC13740466; doi:10.1111/codi.70637)
Supplement: Supplementary file 11 [file CODI-28-0-s011.docx]

# **PERI-SCOPES**

**Peri-Operative Surgical Care Optimisation for Patients requiring Emergency Surgery**

**FAMILY/SUPPORTER CONSENT FORM**	Please initial box

1. I confirm that I have read the information sheet (version...........date……………..…) and privacy notice (version…….date..……) for the above study. I have had the opportunity to consider the information, ask questions and have had these answered satisfactorily.
2. I understand that my participation is voluntary and that I am free to withdraw at any time
   without giving any reason, without my legal rights being affected.
3. I agree to my interview being audio-recorded
4. I understand that the recorded interview will be transcribed word by word and the transcription stored until the study ends in University archiving facilities in accordance with Data Protection policies and regulations.
5. I understand that my information and things that I say in an interview may be quoted in reports and articles that are published about the study, but my name or anything else that could tell people who I am will not be revealed.
6. I understand that data collected during the study, may be looked at by individuals from University of Glasgow, from regulatory authorities or from the NHS Trust, where it is relevant to my taking part in this research.
7. I understand that the information collected about me will be used to support
   other research in the future, and may be shared anonymously with other researchers.
8. I understand and agree to the way my data will be collected and processed and that data will be stored for up to 10 years in University archiving facilities in accordance with Data Protection policies and regulations.
9. If I decide to withdraw, or am withdrawn from the study, I agree that the information collected about me up to the point of my withdrawal will be used in the data analysis for this study.
10. If I do not respond to follow-up requests I give the research team permission to attempt to re-contact me twice (via telephone or post)
11. I agree that my name, contact details and data described in the information sheet will be kept for the purposes of this research project.
12. I agree to take part in the above study.

YES

NO

13. (Optional) I would like to receive a newsletter with the results of the study.
14. (Optional) I am willing for original audio recordings to be used in future	presentations at research conferences. I understand that these recordings	will not be anonymous because they are original audio recordings.

NO

YES

NO

15. (Optional) I am willing for original video recordings to be used in future	presentations at research conferences. I understand that these recordings	will not be anonymous because they are original video recordings.

YES

Name of Participant	Date	Signature

Name of Person	Date	Signature

seeking consent
