# Supplementary material for: Peri‐operative surgical care optimisation for patients requiring emergency surgery (PERI‐SCOPES): Protocol for a multicentre mixed‐methods study
Source: Colorectal Dis. 2026 Nov 5;28(11):e70637. doi: 10.1111/codi.70637 (PMC13740466; doi:10.1111/codi.70637)
Supplement: Supplementary file 12 [file CODI-28-0-s012.docx]

# **PERI-SCOPES**

**Peri-Operative Surgical Care Optimisation for Patients requiring Emergency Surgery**

**CONSULTANT CRF**

**PERI-SCOPES PARTICIPANT ID:**

**Participant Eligibility**

**Inclusion**

- Key decision-makers will be identified in WS1 (predicted: surgeons, intensivists, anaesthetics)
- Post-CCT (Certificate of Completion of Training)
- Participate in active on-call for EGS in a UK-based Hospital where they make decisions in EGS regularly

**Exclusion**

- Consultants not done >2 years of on-call in last 2 years

**Consent**

Date of consent:

Consent version:

Consented by:

| **Baseline Measurements** *(time of recruitment)* | | |  | |
| --- | --- | --- | --- | --- |
| 1.1 | Sex | 🞏 Male | | 🞏 Female |
| 1.2 | Deanery of current practice |  | | |
| 1.3 | Years of experience (consultant) |  | | |
| 1.4 | Sub-speciality |  | | |
