# Supplementary material for: Peri‐operative surgical care optimisation for patients requiring emergency surgery (PERI‐SCOPES): Protocol for a multicentre mixed‐methods study
Source: Colorectal Dis. 2026 Nov 5;28(11):e70637. doi: 10.1111/codi.70637 (PMC13740466; doi:10.1111/codi.70637)
Supplement: Supplementary file 13 [file CODI-28-0-s013.docx]

# **PERI-SCOPES**

**Peri-Operative Surgical Care Optimisation for Patients requiring Emergency Surgery**

**PATIENT CRF**

**PERI-SCOPES PARTICIPANT ID:**

- Patient CRF to be completed by member of research team (research nurse/RJC)
- Baseline measurements: at time of recruitment.
- Assessment 1: within 4 weeks of decision date
- Assessment 2: 3 months after decision date
- Assessment 3: between 9-12 months after decision date
- Questionnaires: within 4 weeks, 3 months, between 9-12 months

**Patient Eligibility**

Inclusion/exclusion criteria should mirror NELA inclusion/exclusion criteria and NELA NoLap guidelines

**Exclusion**

- Age <65 years old
- NoLap patients if management involved interventional radiology or endoscopic procedures
- Patients who are offered a period of conservative treatment are not automatically NoLap
- Adults with diagnosis of dementia or long-standing cognitive impairment.

**Inclusion**

- Age ≥65 years old
- Able to communicate in English
- Cognitively able to complete the questionnaire/interviews
- Able to provide informed, voluntary consent
- First line treatment is expedited, urgent or emergency abdominal surgery on the gastrointestinal tract (would be eligible for inclusion in NELA).
- Surgery can be laparoscopic or open approach.

**Consent Details**

Date of consent:

Consent version:

Consented by:

| **Baseline Measurements** *(time of recruitment)*  Date of Assessment:  Completed by: (Researcher name) | | | |  | |  |
| --- | --- | --- | --- | --- | --- | --- |
| **Section One: Demographics & Basic information** | | | | | |  |
| 1.4 | Sex | | 🞏 Male | | 🞏 Female |  |
| 1.5 | Age at time of recruitment | |  | | |  |
| 1.6 | Scottish Deprivation Score | | Overall rank/Decile | | |  |
| 1.7 | Education level | |  | | |  |
| 1.8 | Ethnicity | |  | | |  |
| 1.9 | Work status | | 🞏 Employed  🞏 Unemployed | | 🞏 Retired |  |
| 1.10 | Pre-admission independence | | 🞏Independent 🞏Family support  🞏Careers | | 🞏Residential home  🞏Care home  🞏Nursing home |  |
| **Section Two: Medical information** | | | | | |  |
| 2.1 | Clinical or radiological diagnosis | |  | |  |  |
| 2.2 | Decision | | 🞏 Emergency operation | |  |  |
|  |  |  | 🞏 No operation | | |  |
| 2.3 | Date of decision | |  | | |  |
|  |  |  |  | | |  |
|  |  |  |  | | |  |
| 2.4 | If operation – what operation?  If no operation – why not? | |  | | |  |
| 2.5 | Consultant specialty involved in decision | | 🞏General Surgeon  🞏Anaesthetist  🞏Critical care / Intensivist  🞏Others: | | |  |
| 2.6 | Charlson co-morbidity index | | Score: | | |  |
|  |  |  | Co-morbidities: | | |  |
| 2.7 | Predicted NELA score |  | | |  |  |
| 2.8 | Rockwood Frailty Score | |  | | |  |
| 2.9 | Pre-admission DNACPR | | 🞏 Yes 🞏 No | | |  |

| **Assessment One** *(within 4 weeks of decision date)*  Date of Assessment:  Completed by: (Researcher name) | | |  | |
| --- | --- | --- | --- | --- |
| 1.1 | Mortality | 🞏 Alive and out of hospital  Number of days:  🞏 Alive and in hospital  🞏 RIP | |  |
| 1.2 | Complications | Clavein-Dindo  🞏Grade 1  🞏Grade 2  🞏Grade 3a  🞏Grade 3b  🞏Grade 4  🞏Grade 5  Details: | |  |
| 1.3 | Length of hospital stay |  | |  |
| 1.4 | Critical care stay | 🞏ward only  🞏HDU  🞏ITU | |  |
| 1.5 | If employed pre-admission | 🞏 Returned to work  🞏 Not returned to work | | 🞏 N/A |
| 1.6 | Level of independence | 🞏Independent 🞏Family support  🞏Careers | | 🞏Residential home  🞏Care home  🞏Nursing home |

| **Assessment Two** *(3 months after decision date)*  Date of Assessment:  Completed by: (Researcher name) | | |  | |
| --- | --- | --- | --- | --- |
| 1.1 | Mortality | 🞏Alive and out of hospital  Number of days:  🞏 Alive and in hospital  🞏 RIP | |  |
| 1.2 | Readmission | 🞏Yes  Number of readmissions:  🞏 No | |  |
| 1.3 | Charlson co-morbidity index | Score: | |  |
|  |  | Co-morbidities: | |  |
| 1.4 | Clinic appointments |  | |  |
| 1.5 | Rockwood Frailty Score |  | |  |
| 1.6 | Level of independence | 🞏Independent 🞏Family support  🞏Careers | | 🞏Residential home  🞏Care home  🞏Nursing home |

| **Assessment Three** *(between 9-12 months after decision date)*  Date of Assessment:  Completed by: (Researcher name) | | |  | |
| --- | --- | --- | --- | --- |
| 1.1 | Mortality | 🞏Alive and out of hospital  Number of days:  🞏 Alive and in hospital  🞏 RIP | |  |
| 1.2 | Readmission | 🞏Yes  Number of readmissions:  🞏 No | |  |
| 1.3 | Charlson co-morbidity index | Score: | |  |
|  |  | Co-morbidities: | |  |
| 1.4 | Clinic appointments |  | |  |
| 1.5 | Rockwood Frailty Score |  | |  |
| 1.6 | Level of independence | 🞏Independent 🞏Family support  🞏Careers | | 🞏Residential home  🞏Care home  🞏Nursing home |

| **Questionnaires** | | |  | |
| --- | --- | --- | --- | --- |
| 1.1 | Within 4 weeks of decision date | Date Sent:  Date Returned: | |  |
| 1.2 | 3 months after decision date | Date Sent:  Date Returned: | |  |
| 1.3 | Between 9-12 months after decision date | Date Sent:  Date Returned: | |  |
